# Complete Inactivation of Blood Borne Pathogen *Trypanosoma cruzi* in Stored Human Platelet Concentrates and Plasma Treated With 405 nm Violet-Blue Light

**DOI:** 10.3389/fmed.2020.617373

**Published:** 2020-11-24

**Authors:** Katarzyna I. Jankowska, Rana Nagarkatti, Nirmallya Acharyya, Neetu Dahiya, Caitlin F. Stewart, Ruairidh W. Macpherson, Mark P. Wilson, John G. Anderson, Scott J. MacGregor, Michelle Maclean, Neil Dey, Alain Debrabant, Chintamani D. Atreya

**Affiliations:** ^1^Laboratory of Cellular Hematology, Office of Blood Research and Review, Center for Biologics Evaluation and Research, Food and Drug Administration, Silver Spring, MD, United States; ^2^Laboratory of Emerging Pathogens, Office of Blood Research and Review, Center for Biologics Evaluation and Research, Food and Drug Administration, Silver Spring, MD, United States; ^3^The Robertson Trust Laboratory for Electronic Sterilization Technologies, Department of Electronic and Electrical Engineering, University of Strathclyde, Glasgow, United Kingdom; ^4^Department of Biomedical Engineering, University of Strathclyde, Glasgow, United Kingdom; ^5^Canary, Inc., Acton, MA, United States

**Keywords:** *Trypanosoma cruzi*, parasite, platelets, plasma, pathogen-reduction technologies, 405 nm light

## Abstract

The introduction of pathogen reduction technologies (PRTs) to inactivate bacteria, viruses and parasites in donated blood components stored for transfusion adds to the existing arsenal toward reducing the risk of transfusion-transmitted infectious diseases (TTIDs). We have previously demonstrated that 405 nm violet-blue light effectively reduces blood-borne bacteria in stored human plasma and platelet concentrates. In this report, we investigated the microbicidal effect of 405 nm light on one important bloodborne parasite *Trypanosoma cruzi* that causes Chagas disease in humans. Our results demonstrated that a light irradiance at 15 mWcm^−2^ for 5 h, equivalent to 270 Jcm^−2^, effectively inactivated *T. cruzi* by over 9.0 Log_10_, in plasma and platelets that were evaluated by a MK2 cell infectivity assay. Giemsa stained *T. cruzi* infected MK2 cells showed that the light-treated parasites in plasma and platelets were deficient in infecting MK2 cells and did not differentiate further into intracellular amastigotes unlike the untreated parasites. The light-treated and untreated parasite samples were then evaluated for any residual infectivity by injecting the treated parasites into Swiss Webster mice, which did not develop infection even after the animals were immunosuppressed, further demonstrating that the light treatment was completely effective for inactivation of the parasite; the light-treated platelets had similar *in vitro* metabolic and biochemical indices to that of untreated platelets. Overall, these results provide a proof of concept toward developing 405 nm light treatment as a pathogen reduction technology (PRT) to enhance the safety of stored human plasma and platelet concentrates from bloodborne *T. cruzi*, which causes Chagas disease.

## Introduction

Chagas disease (CD) is caused by a protozoan parasite, *Trypanosoma cruzi* (*T. cruzi*), which is transmitted to animals and humans by blood-sucking triatomine insects that are mainly found in South America (1). In humans, *T. cruzi* infection occurs by congenital transmission and rarely through blood transfusion. Cases of transmission have also been reported through organ transplantation, accidental blood exposure or by consumption of food contaminated with infected triatomine insects (2). The mammalian life cycle stages of the parasite include trypomastigotes that circulate in the blood-stream, and amastigotes, the replicative form of the parasite located inside mammalian cells (3, 4).

In the first phase of infection, the acute phase, that lasts up to a few weeks or months, *T. cruzi* trypomastigotes can be detected in blood by microscopy or PCR. The majority of individuals in the acute phase have mild or no symptoms. After the acute phase, infected individuals enter a chronic asymptomatic form of disease during which about 30% of the infected individuals go on to develop clinical symptoms associated with CD (5). *T. cruzi* infection is more difficult to detect in the chronic phase as the number of parasites in the blood reduces significantly and are undetectable by microscopy (6).

Worldwide about 8 million people are affected by CD. More than 12,000 deaths are reported each year, associated with chronic heart disease caused by cardiac tissue damage due to *T. cruzi* infection (3, 7). According to the Centers for Disease Control and Prevention (CDC), over 300,000 people are infected with *T. cruzi* in the U.S. (8). Due to the presence of *T. cruzi* trypomastigotes in the blood of infected individuals, in the U.S., the risk of acquiring CD is mostly attributable to blood transfusions by otherwise healthy asymptomatic blood donors. Thus, to reduce the risk of transfusion-transmitted CD, many countries including the U.S., have been screening donated blood for antibodies against *T. cruzi* antigens. In 2010, the U.S. Food and Drug Administration (FDA) recommended one-time testing of each donor of blood and blood components intended for transfusion using a licensed test for antibodies to *T. cruzi* (https://www.fda.gov/BiologicsBloodVaccines/GuidanceComplianceRegulatoryInformation/Guidances/default.htm). However, it is possible that serological tests may miss individuals with low antibody titers, and the results of serological diagnosis may be influenced by antigenic variation across different *T. cruzi* genotypes as well as the genetic background of the donors (9).

To mitigate the risks of transfusion-transmitted infections, several Pathogen Reduction Technologies (PRTs) have been developed or are currently under investigation. In the U.S., the only FDA approved pathogen reduction system for platelets and plasma, the Cerus INTERCEPT pathogen reduction system, uses ultraviolet (UVA) light-induced crosslinking of a psoralen photochemical (Amotosalen) as a nucleic acid intercalator to inactivate pathogens by disrupting their DNA or RNA replication (10). The INTERCEPT system has been demonstrated to reduce *T. cruzi* by >5.4 Log_10_ in platelet concentrates (PCs) and by >5.0 Log_10_ in plasma (PL) (11). Inactivation of *T. cruzi* using the Terumo BCT Mirasol pathogen reduction system, approved for use in other countries, utilizes riboflavin as a photosensitizer in combination with UV light, and has been demonstrated to inactivate 5–7 Log_10_ parasites in PCs and PL (12).

In parallel, numerous studies have also demonstrated that 405 nm violet-blue light which is in the visible spectrum can effectively inactivate a wide range of pathogens including bacteria, spore-forming bacteria, fungi (13–16) and some noroviruses in liquid medium as well as in human plasma (17). This 405 nm treatment for pathogen inactivation has added operational advantages of not requiring the addition of photosensitizers or extensive processing and requiring only direct exposure of the storage container (bag) that has the transfusion component (plasma or platelets suspended in plasma).

To our knowledge, use of 405 nm violet-blue light to reduce important bloodborne protozoan parasites such as *T. cruzi, Babesia microti* or *Plasmodium falciparum* has not yet been evaluated. Toward addressing this goal, as a first step in evaluating the potential of 405 nm light as a PRT for blood-borne parasite inactivation, in this report we investigated the microbicidal potential of the violet-blue light to inactivate *T. cruzi* parasites in PL and PCs. Our results demonstrate that the 405 nm light can completely inactivate all forms of *T. cruzi* to the extent that no residual infectivity was observed in Swiss Webster (SW) mice even after immunosuppression of the mice with three doses of Cyclophosphamide.

## Materials and Methods

### Human Platelets and Plasma

Human PL and PCs were obtained from the National Institutes of Health Blood Bank (Bethesda, MD, USA). Platelets stored at 22 ± 2°C under agitation for 1 day were utilized for the 405 nm light experiments. Plasma was stored either at 4°C overnight (if fresh) or thawed from the frozen stock for use in these experiments. Study involving human subjects' protocol was approved by FDA Research Involving Human Subjects Committee (RIHSC, Exemption Approval #11-036B).

### 405 nm Violet-Blue Light Exposure

All experiments involving the 405-nm light treatment of platelets and plasma were performed in a closed system (US Patent Application no. 62/236, 706, 2015), which contained a light source composed of narrowband 405 nm LED arrays (FWHM ~20 nm; LED Engin, CA, USA), with appropriate thermal management and powered by LED drivers (Mean Well, Taiwan). The light source was held at a distance of 14 cm above the samples.

For 405 nm light exposure of plasma and platelet concentrates spiked with *T. cruzi*, samples were held in T-25 falcon flasks at a volume of 6 ml (*n* = 4). Control experiments for PCs were done in transfer bags (Terumo Transfer Bag T-150; Terumo, Lakewood, CO) with a volume of 40 ml (*n* = 4). The irradiance of 405 nm light across the area of the samples was recorded using the using a radiant power meter and photodiode detector (LOT-Oriel Ltd., USA), and the mean irradiance across the surface was calculated to be approximately 15 mW/cm.

Samples were exposed to 15 mW/cm^2^ 405 nm light for a period of up to 5 h. The exposure dose (Jcm^−2^) was calculated as the product of the irradiance (W/cm^2^) multiplied by the exposure time (sec). Thus, the dose level after 5 h of 405 nm light treatment at an irradiance of ~15 mW/cm^2^, was equal to 270 J/cm^2^. The experimental system was held in a shaker incubator at 72 rpm and 22°C to allow continuous sample agitation and to maintain constant exposure conditions.

### Determination of Platelet Properties

Platelets were counted on the pocH-100i hematology analyzer (Sysmex) before treatment (Ctrl) and after 5 h of 405 nm light exposure (Test 5h)

The biochemical properties of PCs such as pH, lactate and glucose concentration, partial carbon dioxide and oxygen pressure as well as potassium and chloride concentrations before (untreated Ctrl) and after 5 h of light exposure (Test 5h) were compared using a Start Profile CCS Time Blood Gas Analyzer (Nova Biomedical, Waltham, MA, USA).

### Platelet Activation Assay

The level of platelet activation was determined by measuring the expression of p-selectin, a cell membrane marker of platelet activation, by Fluorescence-activated cell sorting (FACS) flow cytometry. FACS analysis was performed using FITC or PE conjugated antibodies against CD41 (Platelet Glycoprotein IIb) and CD62 (p-selectin). CD41-FITC and CD62-PE antibodies were obtained from Beckman Coulter and Becton Dickinson (Becton Dickinson, San Jose, CA), respectively. Isotype controls, Isotype IgG-FITC (Beckman Coulter) and Isotype IgG-PE (Becton Dickinson, San Jose, CA) were used for setting background fluorescence. Platelets were washed with PBS-human albumin solution and resuspended in the same solution. Platelets (1 × 10^6^) were incubated with saturating concentrations of the antibodies and incubated at room temperature in dark conditions. After 20 min incubation, platelets were washed with Phosphate Buffered Saline (PBS)-human albumin solution, resuspended in 200 μl PBS-human albumin solution and analyzed using a FACSCalibur (Becton Dickinson, San Jose, CA) and CellQuestPro software. Data analysis was performed using FlowJo software (Becton Dickinson, San Jose, CA).

### Platelet Apoptosis Assay

Platelet apoptosis was calculated from phosphatidylserine (PS) exposure on platelet surface using PE Annexin V Apoptosis Detection Kit I from BD (Becton Dickinson, San Jose, CA). Platelets were washed with PBS-human albumin solution and resuspended in annexin binding buffer. Platelets (1 × 10^6^) were incubated with saturating concentrations of the Annexin-V and 7AAD antibodies and incubated for 15 min in dark conditions. After incubation, platelets were washed with annexin binding buffer and analyzed using a FACSCalibur (Becton Dickinson, San Jose, CA) and CellQuestPro software. Data analysis was performed using FlowJo software (Becton Dickinson, San Jose, CA).

### Platelet Aggregation Assay

The platelet aggregation was performed in AggRAM (Helena Laboratories, Beaumont, TX USA). Briefly, platelets from control and test samples were diluted to 2.5 × 10^8^/ml in plasma. After optical adjustment with water, aggregation was set to 100% using platelet poor plasma (PPP). Platelet aggregation was measured against two different agonists, collagen and Adenosine 5'-diphosphate using AggRAM manufacturer's protocol; collagen is a strong agonist (aggregation-stimulant) and ADP is a weak agonist.

### *T. cruzi* Culture and TCID_50_ Estimation

To obtain high titers of parasites, MK2 cells (ATCC® CCL-7™) were infected with *T. cruzi* trypomastigote parasites (Colombiana strain). Monolayers of MK2 cells were cultured in DMEM medium containing 10% fetal bovine serum. *T. cruzi* trypomastigotes were incubated with the adherent MK2 cells at 1:10 multiplicity of infection (MoI) for 24 h. The MK2 cells were washed with medium to remove extracellular parasites after 24 h. The infected monolayer was cultured for 4–5 days till extracellular motile trypomastigotes were observed in the medium. Culture supernatants containing trypomastigotes from four T-75 culture flasks were pooled and parasites collected by centrifugation. Prior to 405 nm light treatment, *T. cruzi* trypomastigotes were washed with and resuspended in PBS. Viability was estimated by fluorescent live/dead imaging using a mix of acridine orange and propidium iodide with a Cellometer K2 automated cell counter (Nexcelom, Lawrence, MA) as per manufacturers protocol.

For 405 nm light inactivation studies, parasites were diluted at a concentration ≥ 1 × 10^8^ parasites in 16 ml of human PCs or PL. The diluted parasites were split in equal volumes into two T-25 tissue culture flasks and treated with 405 nm light as indicated above. To estimate the number of parasite surviving after exposure, MK2 cells resuspended in complete DMEM medium at a concentration of 10,000 cells/200 μl was seeded in 96-well tissue culture plates (200 μl per well). The tissue culture plates were incubated for 24 h prior to infection with light exposed *T. cruzi* trypomastigotes.

Aliquots of 200 μl were removed from the light exposed and unexposed flasks at 0, 2, 4, and 5 h post exposure (equivalent to doses of ~108, 216, 270 J/cm^2^, respectively). The Reed and Muench method was used to estimate 50% tissue culture infectious dose (TCID_50_, the dilution at which 50% of the wells are infected) (11). Serial dilutions (1:10) of 2 ml each were prepared from the aliquots in DMEM medium (lowest dilution tested was 10^−1^ and the highest dilution tested, 10^−12^). For each dilution, 100 μl/well was used to infect eight replicate wells of MK2 cells. The plates were cultured for 28 days and wells were scored according to the presence or absence of motile extracellular trypomastigotes visible via light microscopy. Wells with motile trypomastigotes were scored as positive and percent positivity was used to calculate Log_10_TCID_50_. *T. cruzi* inactivation was determined by subtracting the TCID_50_ after exposure from the TCID_50_ of the control unexposed sample for each time point. At least three independent experiments each were performed for *T. cruzi* spiked plasma and platelet concentrates.

### Giemsa Staining

Eight-well chambered glass slides were seeded with 10,000 MK2 cells/well and incubated for 24 h prior to infection with *T. cruzi* trypomastigotes. Viability of light exposed, and unexposed parasites spiked in plasma was determined using the Cellometer as indicated above. Each well of the chambered slide received viable parasites from the 5 h exposed or the control unexposed flask at 1:10 MOI. After a 24-h incubation the chambers were washed with DMEM medium and the slides incubated at 37°C for 6 days. During the 6-day incubation period the wells were observed by light microscopy for the presence of motile extracellular trypomastigotes. On days 3 and 6, slides were washed with PBS and stained with Diff-Quik Giemsa stain as per manufacturers protocol. At least three independent staining experiments were performed for *T. cruzi* parasites spiked in plasma.

### Injection of Blue-Light Treated *T. cruzi* Into Mice and Infection Evaluation

Female Swiss Webster (SW) mice were purchased from Charles River (Wilmington, MA). The animals were used under a protocol approved by the Center for Biologics Evaluation and Research Animal Care and Use Committee (ASP#2010-03). All experiments were performed following relevant guidelines and regulations contained in the 8th edition of The Guide for the Care and Use of Laboratory Animals, National Research Council, 2011 and U.S. Public Health Services Policy on animal welfare. Mice were maintained at the FDA/CBER American Association for the Accreditation of Laboratory Animal Care accredited animal facility under standard environmental conditions for this species.

Mice were infected at 5–7 weeks of age (*n* = 3 mice per group/experiment). As experimental controls, a group of SW mice were infected with 10,000 unexposed trypomastigotes spiked in plasma by intraperitoneally injections. For evaluating the inactivation of blue-light exposed parasites spiked in plasma exposed for 5 h, mice were injected intraperitoneally with 2 × 10^6^ or 4 × 10^6^ trypomastigotes/mouse. Prior to intraperitoneal injections, the parasites were counted using the Cellometer viability stain, collected by centrifugation and washed with PBS to prevent adverse reactions to human PL proteins. Blood parasitemia levels were determined using light microscopy beginning at 15 days post infection (dpi). Blood drawn from the tail vein (5 μl drop) was mounted between a microscope slide and cover slip and the number of motile trypomastigotes was estimated as described previously (6, 18). Blood parasitemia was also estimated in the chronic phase of infection (4–6 months after infection). Two independent experiments were performed in SW mice.

All mice were treated with cyclophosphamide (CY), a potent immunosuppressant which was administered at 10 mg/kg body weight per injection for four consecutive days. Mice were observed for 3 days and on the 3rd day tail vein bleeds were performed to detect blood parasitemia by light microscopy. A total of three cycles over a period of 21 days were performed and mice were euthanized following the last cycle (19).

### Statistical Analysis

Statistical analysis was performed using Microsoft Excel and Prism software. Unless otherwise indicated, statistical significance was calculated using Student's T test for unpaired samples, and data are presented as mean ± standard deviation (SD), at the 95% confidence level.

## Results

### 405 nm Violet-Blue Light Exposure Dose

In our previous studies we investigated bacterial inactivation rates under different exposure doses ranging from 36 to 288 J/cm^2^. PCs exposed to 10 mW/cm^2^ light for 8 h (288 J/cm^2^) and injected into SCID mice were found to sustain the same recovery as untreated PC samples (16). In this study, we have applied a higher irradiation of 15 mW/cm^2^ light but for a shorter time, up to 5 h, equivalent to a reduced dose of 270 Jcm^−2^. Doses in this region were sufficient to inactivate ≥99% of tested bacteria and virus from human platelets and plasma (16).

### Platelet Properties After 405 nm Light Exposure

**Figure 1:**
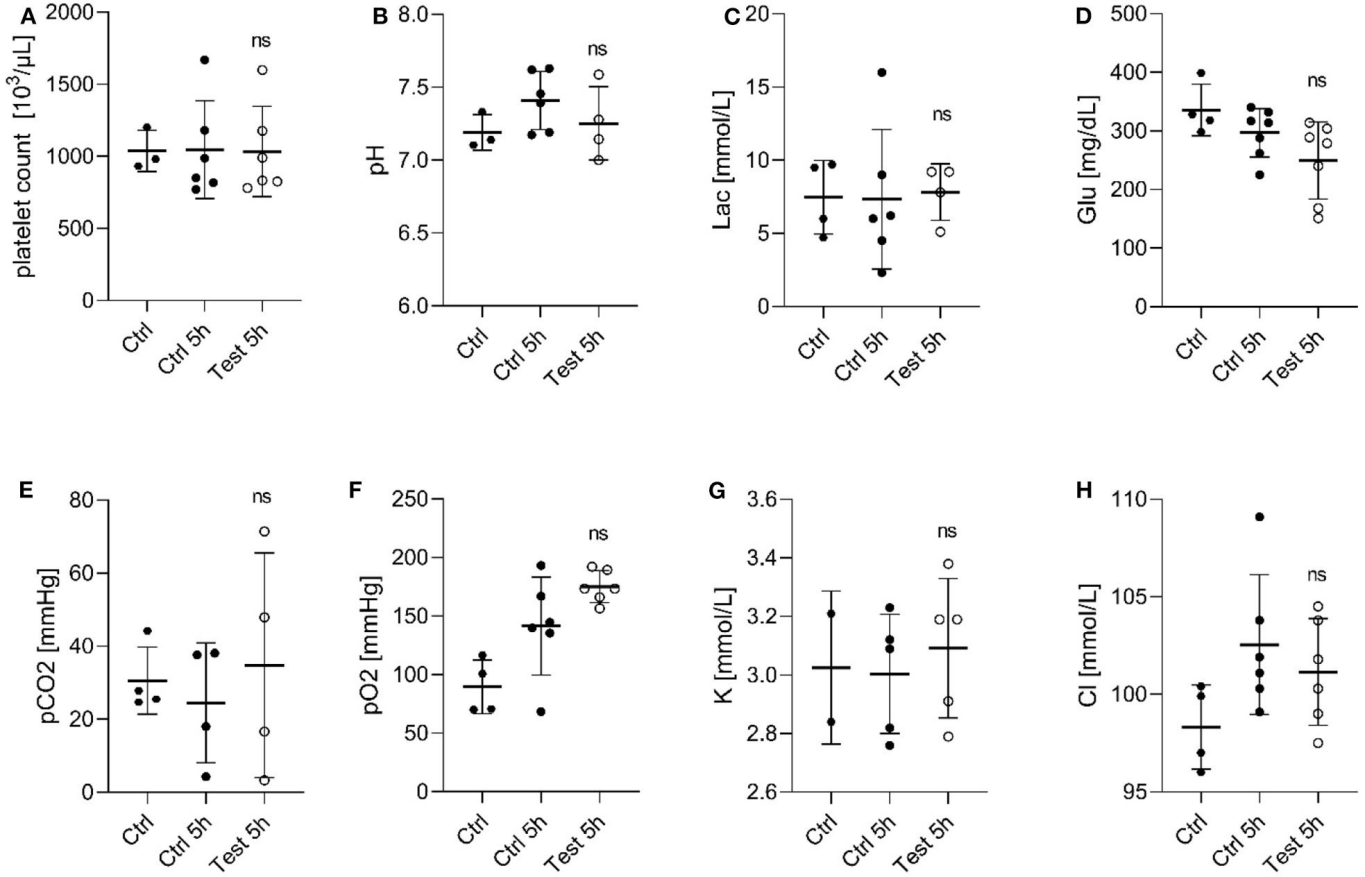
Effect of 405 nm light exposure on platelet parameters after exposure to 15 mW/cm^2^ for 5 h (270 Jcm^−2^) (Test_5h) compared to control platelets before (Ctrl) and after 5 h in shaker (Ctrl_5h) **(A)** Platelet count (Mean ± SD: Ctrl = 1,037 ± 142.9, Ctrl 5h = 1,046 ± 339.3, Test 5h = 1,034 ± 313.0); **(B)** pH of platelet concentrates (Mean ± SD: Ctrl = 7.191 ± 0.122, Ctrl 5h = 7.411 ± 0.199, Test 5h = 7.253 ± 0.251); **(C)** Lactate level [Lac (mmol/L), Mean ± SD: Ctrl = 7.475 ± 2.512, Ctrl 5h = 7.333 ± 4.781, Test 5h = 7.825 ± 1.933]; **(D)** Glucose level [Glu (mg/dL), Mean ± SD: Ctrl = 335.8 ± 43.97, Ctrl 5h = 296.9 ± 41.34, Test 5h = 249.3 ± 65.826]; **(E)** Partial carbon dioxide pressure [pCO_2_ (mmHg), Mean ± SD: Ctrl = 30.55 ± 9.19, Ctrl 5h = 24.50 ± 16.40, Test 5h = 34.80 ± 30.74]; **(F)** Partial oxygen pressure [pO_2_ (mmHg), Mean ± SD: Ctrl = 89.53 ± 23.00, Ctrl 5h = 141.5 ± 41.72, Test 5h = 175.2 ± 13.66]; **(G)** Potassium concentration [K (mmol/dL), Mean ± SD: Ctrl = 3.025 ± 0.262, Ctrl 5h = 3.0045 ± 0.2033, Test 5h = 3.092 ± 0.238]; and **(H)** Chloride level [Cl (mmol/dL), Mean ± SD: Ctrl = 98.33 ± 2.156, Ctrl 5h = 102.6 ± 3.576, Test 5h = 101.2±2.733]; Each point represents the data from platelets obtained from different donors measured in at least three independent experiments. The 405 nm exposed samples (Test_5h) relative to the control (Ctrl_5h) were analyzed by *t*-test. ns- not significantly different, at the 95% confidence level.

Next, we evaluated the effect of a 405 nm light dose of 270 Jcm^−2^ on PC by assessing the quality of platelets after 5 h exposure compared to the untreated platelets (Figure 1). It is known that during storage, platelets generate metabolites, such as lactate which cause a decrease in pH leading to morphological changes, and loss of platelet viability *in vivo* (20). A pH below 6.8 is associated with morphological/physiological changes in platelets (21). While at a pH lower than 6.3, platelet function and discoid morphology is lost, and they become more spherical with multiple pseudopods (22). As seen in Figure 1A, platelet counts in PCs remained stable for 5 h of 405 nm light exposure. Similarly, the pH values of PC were within the range 7.0–7.7 in all tested samples (Figure 1B), indicating similar lactate concentrations in treated and untreated platelets. In addition, the glucose level did not decrease drastically during storage or light exposure (Figure 1D). The observed glucose concentration in light exposed platelets was within a range that was also observed in PC during storage (20). The other measured parameters such as partial carbon dioxide and oxygen pressure, or potassium and chloride concentration, were also not significantly affected by 405 nm light exposure (Figures 1E–H).

**Figure 2:**
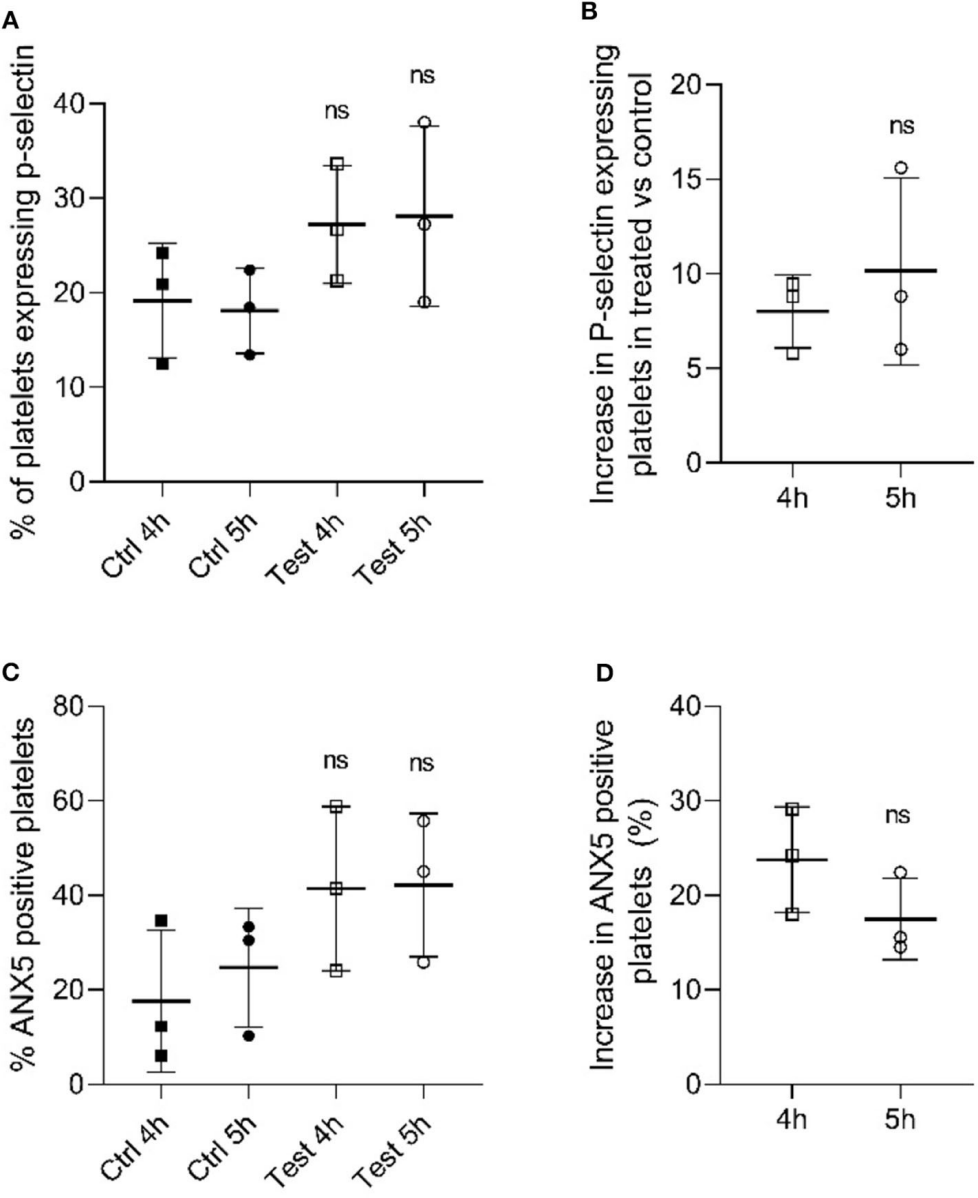
Effect of 405 nm light exposure on platelets. Samples were exposed to 15 mW/cm^2^ for 4 h (216 J/cm^2^) and 5 h (270 J/cm^2^) **(A)** P-selectin expression in platelets after 4 and 5 h exposure at 405 nm (test) vs. unexposed samples (Ctrl) (Mean ± SD: Ctrl 4h = 19.18 ± 6.06, Ctrl 5h = 18.08 ± 4.51, Test 4h = 27.20 ± 6.22, Test 5h = 28.08 ± 9.53). **(B)** Increase in P-selectin expressing cells in tested samples vs. control (Mean ± SD: 4 h = 8.02 ± 1.95, 5 h = 10.13 ± 4.94). **(C)** % ANX5 positive platelets after 4 and 5 h exposure at 405 nm (test) vs. unexposed samples (Ctrl) (Mean ± SD: Ctrl 4h = 17.66 ± 15.00, Ctrl 5h = 24.70 ± 12.55, Test 4h = 41.42 ± 17.38, Test 5h = 42.18 ± 15.12). **(D)** Increase in ANX5 positive platelets in tested samples vs. control (Mean ± SD: 4 h = 23.76 ± 5.58, 5 h = 17.48 ± 4.29). Each point represents the data from platelets obtained from different donor measured in at least 3 independent experiments. The 405 nm exposed samples (test) relative to the control (Ctrl) was analyzed by *t*-test. *p* < 0.05; ns- not significantly different, at the 95% confidence level.

Once we identified that the metabolic parameters of PCs evaluated were within the acceptable ranges following 405 nm light treatment, we next determined if platelet functions were disturbed after illumination with 405 nm-light at a dose of 270 Jcm^−2^. As p-selectin is essential for platelet activation we evaluated the p-selectin expression level in platelets after 4 h (216 J/cm^2^) and 5 h (270 J/cm^2^) light treatment (23). As shown in Figure 2A, platelets exposed to 405 nm light exhibited no significant increase in p-selectin expression compared to unexposed platelets (*P* = 0.185 and *P* = 0.175 for 4 and 5 h, respectively). The increase observed after 4 h was comparable to that observed after 5 h (*P* = 0.528, Figure 2B). Overall, since p-selection expression increases when platelets are activated, we can conclude that 405 nm light did not stimulate platelet activation significantly.

In order to determine the apoptosis level in light-treated and untreated platelets, we evaluated the levels of annexin 5 (ANX5) in PC after exposure to 405 nm light for 4 h (216 J/cm^2^) and 5 h (270 J/cm^2^) (24). As shown in Figure 2C, the level of ANX5 positive platelets, indicative of apoptosis was about 20% higher compared to controls (*P* = 0.528, Figure 2D). However, comparison at an individual sample level demonstrated that two out of three light treated samples tested were within the same range, i.e., not significantly increased (*P* = 0.148 and *P* = 0.198 for 4 and 5 h, respectively).

**Figure 3:**
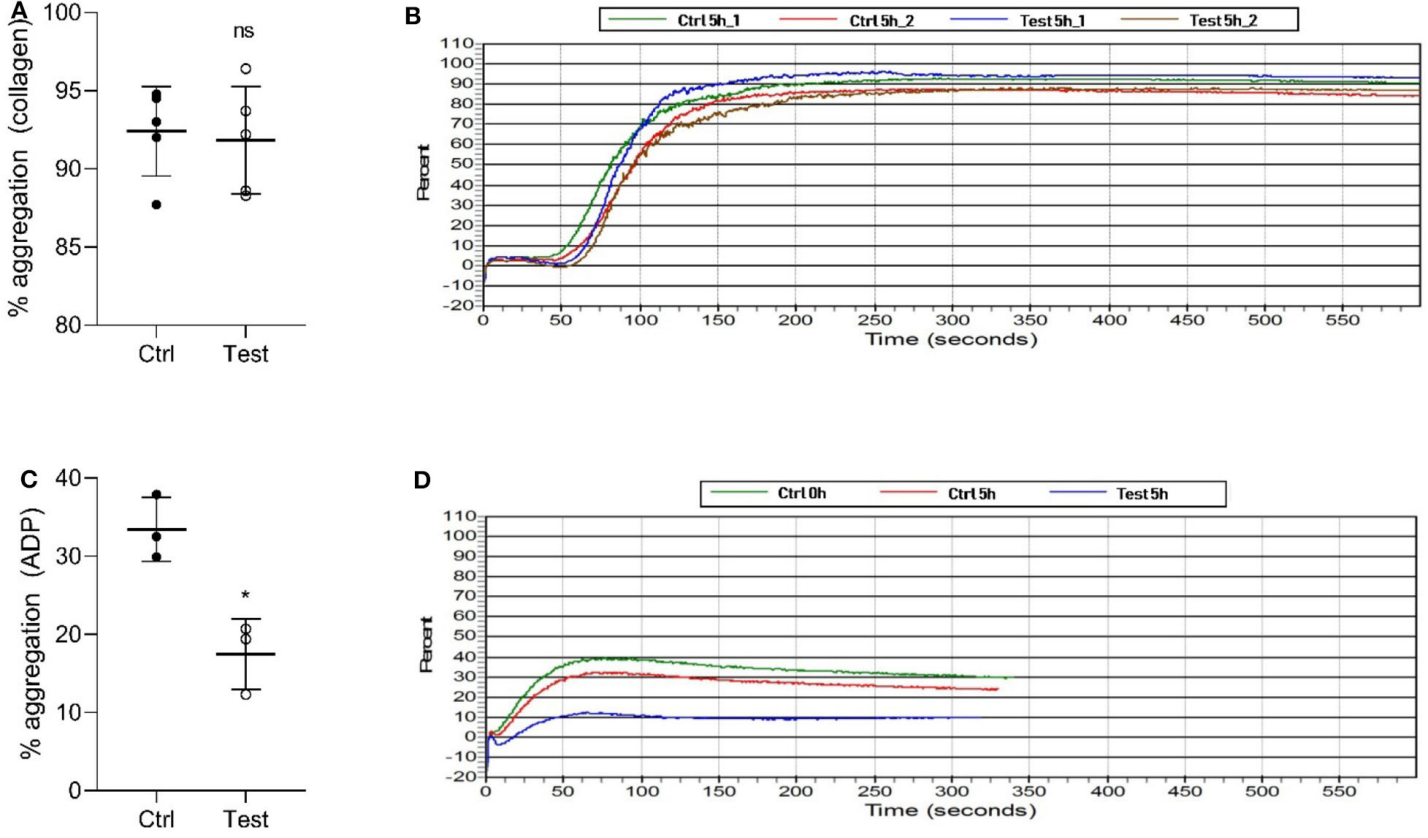
Effect of 405 nm light exposure on platelet aggregation. Samples were exposed to 15 mW/cm^2^ for 5 h (270 J/cm^2^). **(A)** % of platelets aggregation measured by collagen (Mean ±SD: Ctrl = 92.40 ± 2.86, Test = 91.84 ± 3.44) and **(B)** the representative platelet aggregation report. **(C)** % of platelets aggregation measured by ADP (Mean ±SD: Ctrl = 33.43 ± 4.08, Test = 17.47 ± 4.52) and **(D)** the representative platelet aggregation report. Each point in **(A,C)** represents the data from platelets obtained from different donors measured in at least three independent experiments. The 405 nm exposed samples (Test) relative to the control (Ctrl) was analyzed by *t*-test. *p < 0.05; ns- not significantly different, at the 95% confidence level.

We also investigated if 405 nm light affects *in vitro* platelet aggregation (Figure 3). Collagen, a strong agonist, was able to stimulate aggregation in the 270 J/cm^2^ light-treated platelets like in the control group (*P* = 0.7868, Figures 3A,B). However, ADP, a weak agonist, was unable to stimulate aggregation in the light-treated platelets to the levels observed in the control samples (*P* < 0.0105, Figures 3C,D). While collagen and ADP are the two commonly used agonists to evaluate platelet activation, collagen-induced platelet aggregation is the one that occurs under physiological conditions (25). Taken together, the results show that the light treatment if at all, has only a minor effect on platelet quality in terms of aggregation potential relevant to physiological conditions.

### 405 nm Light Inactivates *T. cruzi* trypomastigotes in Plasma and Platelet Concentrates

Blood stage *T. cruzi* trypomastigotes obtained from *in vitro* cultures were spiked into human plasma and platelet concentrates received from individual donors at concentrations >10 × 10^8^ parasites/ml based on Cellometer counts. Exposure of the parasites to blue-light resulted in progressive loss of motility over the course of the experiment as observed by light microscopy. However, the morphology of the trypomastigotes remained intact even at the 5 h time point. As the blue-light has been shown to interact with heme and porphyrin containing proteins (26, 27) and many of them are part of the electron transport chain in the mitochondrial membrane, critical functions of this organelle such as energy production (ATP) are expected to be compromised that result in the loss in motility of the parasite.

**Figure 4:**
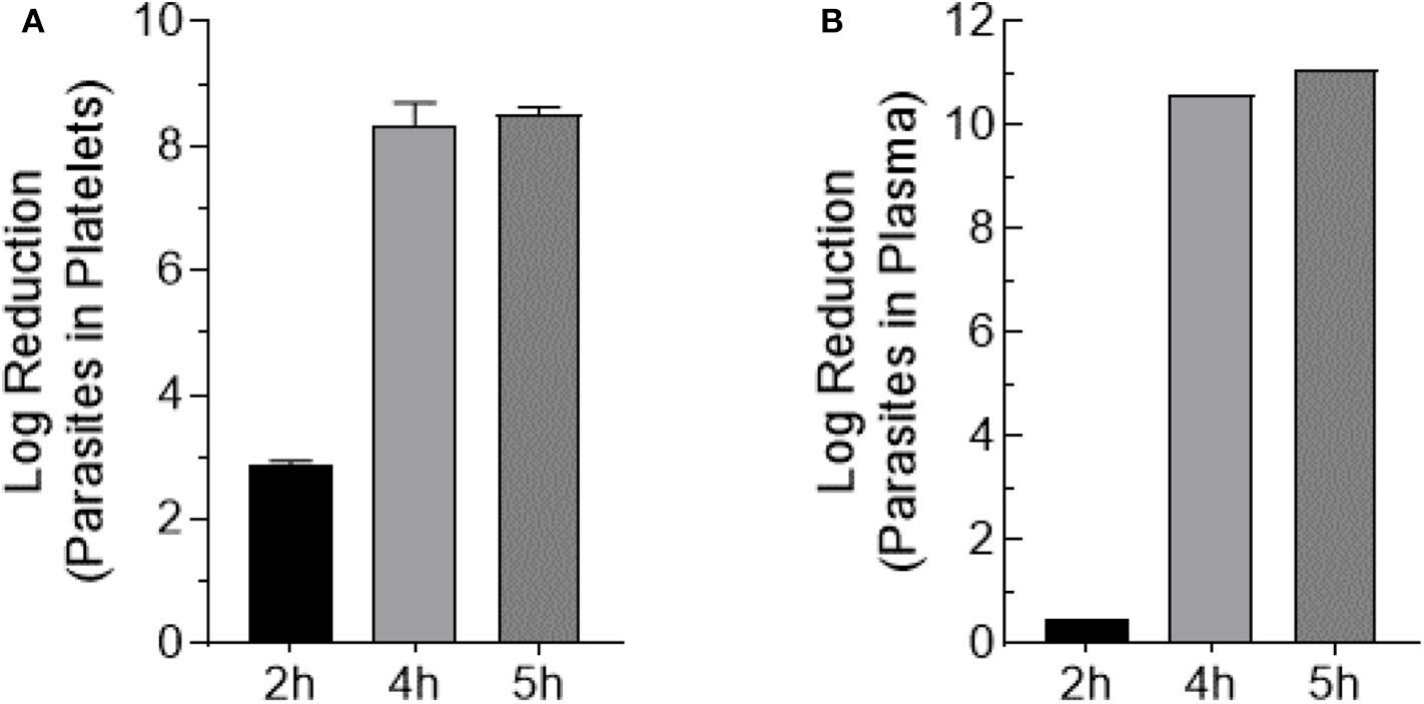
Trypomastigote inactivation by 405 nm blue-light in plasma and platelets. Samples were exposed to 15 mW/cm^2^ for 2 h (108 J/cm^2^), 4 h (216 J/cm^2^), and 5 h (270 J/cm^2^) **(A)** Log reduction values of *T. cruzi* trypomastigotes exposed in PC (Mean ± SD: 2 h exposure = 2.9 ± 0.064, 4 h exposure = 8.4 ± 0.35, 5 h exposure = 8.5 ± 0.12). **(B)** Log reduction values of *T. cruzi* trypomastigotes exposed in plasma (2 h exposure = 0.50, 4 h exposure = 10.60, 5 h exposure = 11.10). Each point represents the data from platelets obtained from at least two independent experiments. For the parasites exposed to 405 nm light in plasma, the mean and SD values for log reduction could not be calculated because at the highest dilution (10^−12^) of the unexposed parasites tested in culture, >50% of the wells showed *T. cruzi* trypomastigote and were scored as positive wells. The titer was estimated to be ≥11 Log_10_TCID_50_.

Trypomastigote-spiked plasma and platelets were exposed to 405 nm light for 0, 2, 4, and 5 h (0, 108, 216, 270 J/cm^2^, respectively). Aliquots were withdrawn at each time point, serially diluted in culture medium and 100 μl/well of each dilution was plated in 8-wells of a 96-well plate previously inoculated with MK2 host cells, for a total volume of 0.8 ml of each dilution. Parasite inoculated plates were observed by microscopy for a period of 28 days. None of the wells inoculated with the blue-light exposed parasites, for either the parasites spiked in platelets or plasma, yielded extracellular motile trypomastigotes. The Log_10_TCID_50_ for unexposed parasites spiked in either platelets or plasma were calculated to be ≤ 8.75 ± 0.53 and ≤ 10.76 ± 0.29, respectively. For sample dilutions where no live parasites were observed, the lower limit of detection was estimated based on the total volume tested (0.8 ml; 0.1 ml × 8-wells) and the lowest dilution (10^−1^) tested. The lower limit of detection was estimated to Log_10_TCID_50_ < 1.0. These results indicated that blue-light exposure resulted in the complete abolition of the ability of *T. cruzi* trypomastigotes to infect or grow in MK2 cells *in vitro*. Due to the complete lack of growth with treated parasites after 28 days of culture, log reduction was calculated as the average input titer calculated for the unexposed parasites for each time point in platelets and plasma (Figures 4A,B).

**Figure 5:**
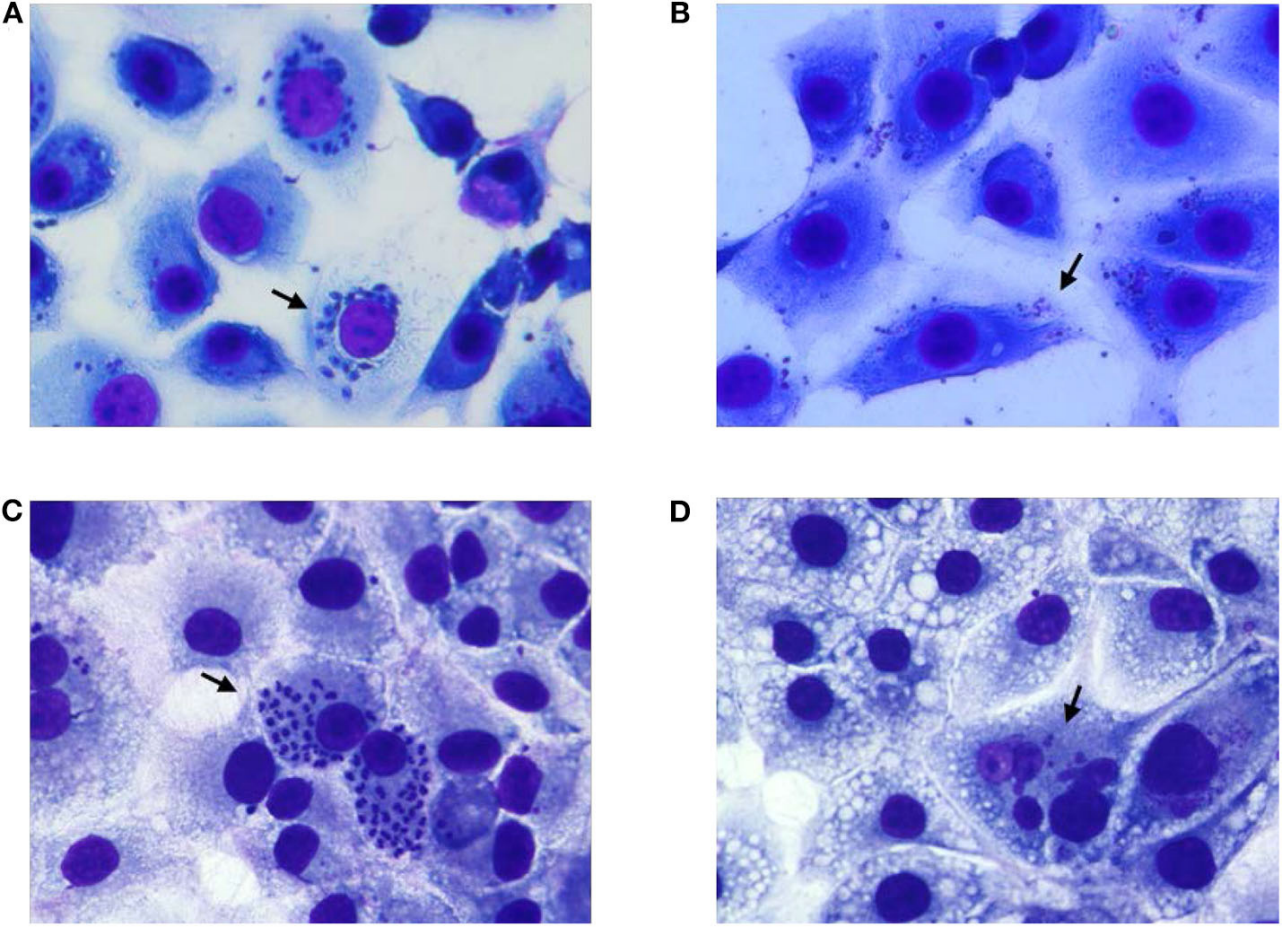
Growth evaluation of *T. cruzi* amastigotes in MK2 cells inoculated with unexposed and 405 nm light exposed *T. cruzi* trypomastigotes. **(A,C)** represent the cells grown on tissue culture slides inoculated with unexposed *T. cruzi* trypomastigotes at 3 days and at 6 days post inoculation, respectively, demonstrating the replication of the parasite inside infected MK2 cells. **(B,D)** represent the cells inoculated with *T. cruzi* trypomastigotes exposed to 15 mW/cm^2^ 405 nm light for 5 h (270 J/cm^2^), at 3 and 6 days post inoculation, respectively, demonstrating the inability of the parasites to replicate. Black arrows represent *T. cruzi* amastigotes within MK2 cells.

To test the complete abolition of the ability of 405 nm light exposed *T. cruzi* trypomastigotes to grow in cell culture, MK2 cells cultured on microscopic slides were inoculated with plasma containing 5 h (270 J/cm^2^) light-treated or untreated parasites at 1:10 MOI and Giemsa stained at different days post inoculation. At day 3 post inoculation, MK2 cells inoculated with treated and untreated parasites showed similar levels of amastigotes. However, Giemsa staining clearly indicated that the untreated parasites within MK2 cells were morphologically better organized compared to the treated parasites within the infected cells (Figures 5A,B, respectively). By day 6 post-inoculation, this phenomenon was clearly distinguishable and while both the treated and untreated parasites were able to enter the cells, treated parasites were unable to replicate further, demonstrating the inactivation of the parasites by the treatment (Figures 5C,D, respectively).

### 405 nm Light Treated *T. cruzi* trypomastigotes Unable to Infect Mice

A SW- *T. cruzi* Colombiana mouse model of Chagas disease was utilized to further assess the viability of the light exposed trypomastigotes. Prior to injecting the parasites into the mice, cell viability of the 5 h light exposed, and unexposed *T. cruzi* trypomastigotes in plasma were estimated using an automated cell counter. In the control group, all the mice injected with 10,000 parasites unexposed to 405 nm-light showed positive parasitemia by 15 dpi. These mice also showed peak parasitemia at 23 dpi in the acute phase of the infection as reported previously for the C57BL/6 -*T. cruzi* Colombiana infection model (18). In the test group, 405 nm light treated trypomastigotes were injected into the mice at a 200- to 400-fold higher number (2 × 10^6^ or 4 × 10^6^) compared to the control group. Injecting a higher number of light-treated trypomastigotes into mice could be considered a worst-case scenario as an equivalent number of unexposed parasites would be lethal to these mice. However, no parasitemia was detectable during the acute phase of infection in the mice injected with 2 × 10^6^ or 4 × 10^6^ trypomastigotes exposed to 405 nm light for 5 h (270 J/cm^2^) (Table 1), suggesting that the treated trypomastigotes were inactivated and unable to replicate and grow in the mice as observed in MK2 cells (Figure 5).

**Table 1:** Average parasitemia estimated by microscopy and % positivity in mice infected with untreated or treated with 405 nm light (270 J/cm^2^).

**Treatment**	**Inoculum (p/mouse)**	**Average p/ml (% positive) at 15 dpi**	**Average p/ml (% positive) at 30 dpi**	**Average p/ml (% positive) post-CY at 180 dpi**	**Average p/ml (% positive) post-CY at 185 dpi**	**Average p/ml (% positive) post-CY at 190 dpi**
Untreated	1 × 10^4^	1.42 × 10^5^ (33%)	1.82 × 10^5^ (100%)	0.1 × 10^5^ (33%)	0.37 × 10^5^ (100%)	n.d.
405 nm Treated	2 × 10^6^	0 (0)	0 (0)	0 (0)	0 (0)	0 (0)
405 nm Treated	4 × 10^6^	0 (0)	0 (0)	0 (0)	0 (0)	0 (0)

Although the light-exposed parasites did not cause any parasitemia and were unable to replicate and grow in mice, to further confirm that there was no residual infectivity hidden in the light-treated parasites, the mice were subjected to immunosuppression. Immunosuppression using Cyclophosphamide has been utilized as a very sensitive method to detect residual parasitemia in chronically infected mice. In the control group infected with unexposed parasites, all the mice showed high levels of blood parasitemia (>1 × 10^4^ parasites/ml) after the second dose of Cyclophosphamide (Table 1). In contrast, in the test group, none of the mice showed blood parasitemia even after three rounds of immunosuppression. This proved beyond doubt that the 405 nm light exposed parasites were completely inactivated.

## Discussion

The introduction of pathogen reduction technologies (PRTs) to treat donated blood components to inactivate multiple types of pathogens (bacteria, fungi, viruses and parasites) with one treatment is a welcome addition to the existing arsenal of donor deferral strategies and serological/nucleic acid testing to reduce the risk of transfusion-transmitted infectious diseases (TTIDs) (28). Pathogen inactivation in stored blood has been attempted since the early 1950's, where use of electron chain inhibitors such as Crystal Violet, Methylene Blue and Toluidine Blue O (TBO), was demonstrated to inactivate parasites (29, 30).

Current PRTs reduce blood-borne pathogens to different degrees, however, the addition of photochemicals in some technologies and irradiation of PCs and PL with different wavelengths of UV light have been well recognized to negatively impact the quality of the treated blood components (31, 32). Therefore, there is still room for improving the existing PRTs and to identify novel second-generation PRTs that are highly effective at pathogen inactivation and show minimal impact on the quality and functionality of the inactivated blood components, that are simple to implement, and relatively inexpensive as envisioned recently (33).

We have previously demonstrated that violet-blue light of 405 nm wavelength alone with no added photosensitizers is effective as a bacterial inactivator (16, 26); since the light wavelength is in the visible spectra, it also served as a gentle treatment for stored platelets in terms of their survival and recovery as evaluated in a SCID mouse model, while providing safety from bacteria (16). In this system, photons at 405 nm excite endogenous porphyrins (circumventing the need for an external photosensitizer) within microbial cells to a higher energy state by energy transfer; subsequent transition of electrons from the porphyrin's excited state to the ground state is accompanied with the release of toxic reactive oxygen species, including singlet oxygen molecules, which cause damage to the microbial membranes, proteins and nucleic-acids resulting in pathogen inactivation (13, 34, 35).

In this report we have evaluated the effect of 405 nm light on platelet metabolic indices and found that these indices are within the range relative to the controls, and also evaluated the aggregation potential of platelets by using collagen, a physiologically relevant strong agonist of platelets and ADP, a weak agonist; the light treatment of platelets did not significantly affect their aggregation potential by collagen. However, ADP could not stimulate aggregation in the light-treated platelets. Similar observations were reported by other investigators (36, 37).

To further evaluate the pathogen inactivation effects of 405 nm blue light, we tested its ability in inactivating *T. cruzi*, the etiological agent of Chagas disease. All cases of transfusion transmitted Chagas disease in the U.S. have been associated with contaminated platelets and a robust method to inactive this parasite would improve blood safety (38, 39). In this report, we demonstrated that *T. cruzi* trypomastigotes can be effectively inactivated by over 9.0 log_10_ in PL and in PCs using a light dose of 270 Jcm^−2^ (15 mWcm^−2^ for 5 h). In this context, it is important to note that other PRT systems have so far been demonstrated to reduce *T. cruzi* by only 5–7 Log_10_ using *in vitro* cell culture methods (11). In contrast to previous cell culture-based studies, here in addition to the cell culture systems, we have also demonstrated that *T. cruzi* is completely inactivated by this level of light treatment using SW mice *T. cruzi* Colombiana infection model. Using this mouse model, we demonstrated that injection of up to 4 × 10^6^ parasites exposed 405 nm light dose in Swiss mice does not result in an infection. This inoculum represents a 400-fold excess of parasites compared to the mice injected with the unexposed parasites (1 × 10^4^/mouse) and would be lethal if not inactivated (40). More significantly, complete inactivation of the 405 nm light-exposed parasites was achieved and demonstrated because, even after immunosuppression with Cyclophosphamide, mice injected with the light-exposed parasites showed no blood parasitemia.

In this context, it is important to note that although 5 h treatment that is reported here appears to be long, this treatment time is much less compared to an already approved pathogen reduction system as there are no downstream processing of the product in case of 405 nm light system. For example, Intercept system which utilizes UV light and Amotosalen for pathogen inactivation, subsequent to the inactivation treatment step, downstream processing includes holding the product with a compound adsorption devise (CAD) to remove Amotosalen from the product for about 4–24 h depending on the type of platelet storage media used [Package Insert, Page 8: INTERCEPT Blood System for Platelets -Small Volume (SV) Processing Set, https://intercept-usa.com/images/resources/Package_Inserts/INTERCEPT_Blood_System_SV_Platelets_Package_Insert_May-2019.pdf].

Since our results provided a proof of concept that an important parasite (*T. cruzi*) relevant to human blood safety can be completely inactivated in PCs and PL using one type of plastic bags, future studies will include comprehensive evaluation of 405 nm light penetration characteristics of different plastic bags as it is well established in the field that for each type of plastic bag used, the light dosing must be calibrated for optimal pathogen inactivation. Future studies also include 405 nm light inactivation potential of *Plasmodium* and *Babesia* parasites that target human red blood cells.

In conclusion, we demonstrated that the blood borne pathogen *T. cruzi* can be effectively inactivated by over 9.0 log_10_ in PL and in PCs using 405 nm light at a dose of 270 Jcm^−2^ (15 mWcm^−2^ for 5 h) and that under these light conditions, platelets retained acceptable metabolic indices and aggregation potential. All the experimental evidences that we have accumulated so far (16, 17, 26), including this report, together form the basis for generating a working hypothesis that 405 nm light treatment could evolve as a simple and gentler but effective PRT system for blood component safety from bacteria, viruses and parasites, provided in-depth studies are under taken in a timely fashion to further evaluate the system.

## Data Availability Statement

The original contributions presented in the study are included in the article/supplementary materials, further inquiries can be directed to the corresponding author/s.

## Ethics Statement

The studies involving human participants were reviewed and approved by FDA Research Involving Human Subjects Committee (RIHSC, Exemption Approval #11-036B). The patients/participants provided their written informed consent to participate in research studies as per NIH Blood Bank ethics guidelines and policies, which supplied human plasma and platelets used in this study. The animal study was reviewed and approved by the Center for Biologics Evaluation and Research Animal Care and Use Committee (ASP#2010-03), FDA. All experiments were performed following relevant guidelines and regulations contained in the 8th edition of The Guide for the Care and Use of Laboratory Animals, National Research Council, 2011 and U.S. Public Health Services Policy on animal welfare.

## Author Contributions

KJ and RN helped in the design of the study, performed data analyses, and participated in writing of the manuscript. NA participated in *T. cruzi* culturing and in mice studies. NDa performed platelets and plasma quality control studies and assisted in the review and editing of the manuscript. AD and CA designed the research plan, oversaw the project, and wrote the manuscript. CS, JA, SM, MM, RM, and MW designed and constructed the 405 nm light device used in this work and participated in writing and editing the manuscript. NDe has participated in writing the manuscript and supported the study. All authors contributed to the article and approved the submitted version.

## Conflict of Interest

A patent is pending on the blue light apparatus used in this study (US Patent Application no. 62/236, 706, 2015). The CBER, FDA has a Cooperative Research and Development Agreement (CRADA) with Canary Inc., USA related to this work and CA is the PI of this work. NDe is an employee of Canary Inc. The remaining authors declare that the research was conducted in the absence of any commercial or financial relationships that could be construed as a potential conflict of interest.
